# Sea urchin reproductive performance in a changing ocean: poor males improve while good males worsen in response to ocean acidification

**DOI:** 10.1098/rspb.2019.0785

**Published:** 2019-07-24

**Authors:** Kathryn E. Smith, Maria Byrne, Dione Deaker, Cameron M. Hird, Clara Nielson, Alice Wilson-McNeal, Ceri Lewis

**Affiliations:** 1 College of Live and Environmental Sciences, University of Exeter, Geoffrey Pope Building, Stocker Road, Exeter EX4 4QD, UK; 2 Schools of Medical and Biological Sciences, University of Sydney, Sydney, New South Wales 2006, Australia

**Keywords:** spermatozoa, individual variability, computer-assisted sperm analysis, fertilization success, echinoderm, carbon dioxide

## Abstract

Ocean acidification (OA) is predicted to be a major driver of ocean biodiversity change. At projected rates of change, sensitive marine taxa may not have time to adapt. Their persistence may depend on pre-existing inter-individual variability. We investigated individual male reproductive performance under present-day and OA conditions using two representative broadcast spawners, the sea urchins *Lytechinus pictus* and *Heliocidaris erythrogramma.* Under the non-competitive individual ejaculate scenario, we examined sperm functional parameters (e.g. swimming speed, motility) and their relationship with fertilization success under current and near-future OA conditions. Significant inter-individual differences in almost every parameter measured were identified. Importantly, we observed strong inverse relationships between individual fertilization success rate under current conditions and change in fertilization success under OA. Individuals with a high fertilization success under current conditions had reduced fertilization under OA, while individuals with a low fertilization success under current conditions improved. Change in fertilization success ranged from −67% to +114% across individuals. Our results demonstrate that while average population fertilization rates remain similar under OA and present-day conditions, the contribution by different males to the population significantly shifts, with implications for how selection will operate in a future ocean.

## 1. Introduction

Ocean acidification (OA) is expected to become one of the greatest drivers of global ocean biodiversity change [1]. Atmospheric CO_2_ levels surpassed 400 ppm in 2016 and have subsequently risen by an additional 5 ppm yr^–1^, above the previously projected increase (2.73 ppm yr^–1^, Keeling curve, Scripps Institute of Oceanography, accessed June 2018) [2]. Due to CO_2_ uptake, the mean pH of ocean surface water has decreased from 8.13 to 8.05 since the industrial revolution and is predicted to drop a further 0.14 to 0.40 units by 2100 (RCP2.6, 8.5) [3]. Seasonal and diurnal variability in pH is also predicted to increase, particularly in coastal areas, suggesting many species may experience greater and more variable extremes in carbonate chemistry [4]. Sensitive marine taxa may not have time to adapt to these rapid changes through new genetic mutations and so the persistence of many species may be determined by the inter-individual genetic variability or plasticity that already exists within populations [5]. Variability in sperm traits both within and among males is well recognized across animal taxa (reviews: [6,7]). Despite this, most studies investigating the impacts of OA on marine taxa have focussed on population-level responses which do not account for individual variability [8–11]. These studies indicate the general sensitivity of a population but fail to identify how inter-individual variability could influence selection and potential longer-term adaptability of a population [5]. In particular, understanding how individuals respond to environmental change may be critical for determining the adaptive potential of populations globally [12,13].

Changes in ocean chemistry have been shown to influence almost every phase of marine invertebrate life cycles, although the reported extent to which different life-history processes are affected vary [10,14–16]. Regardless, while physiological performance in later life may strongly influence the adaptability of a population, early life-history processes such as fertilization, larval development and metamorphosis, are widely understood to be the most vulnerable of life-history stages and are the critical stage for species survival [16–19]. Most marine invertebrates reproduce via broadcast spawning, releasing gametes into the water column for fertilization [20]. Organisms with this mode of reproduction may be particularly susceptible to environmental change because their gametes are exposed to anthropogenic stressors present in the environment prior to fertilization. These stressors can damage or disrupt sperm performance and fertilization processes [21]. In addition, the stress history of the male can influence sperm performance through epigenetic modifications [22,23].

Recent modifications of classic sperm theories, including both theoretical and empirical data, have identified sperm phenotypic traits that are beneficial for fertilization [24–26]. For example, fast swimming speeds have been identified to increase the likelihood of sperm reaching an oocyte first, while longevity gives sperm a greater amount of time to locate an oocyte to fertilize [24,27]. Reported changes in sperm parameters in response to CO_2_-induced acidified seawater are typically negative and most studies identify a reduction in sperm swimming speeds and motility in response to OA [5,10,11,28,29]. By contrast, a few studies report either a neutral or stimulatory response [15,30]. There is no clear trend on the impacts of OA on fertilization success in marine invertebrates [8,10,31], despite sperm traits including high swimming velocity being linked to fertilization efficiency [27,29]. The differences between studies might in part be explained by inter-individual male variability in the response of sperm traits to OA within a population. By looking at individual responses, we may begin to tease apart how OA influences selection across marine invertebrates and achieve a greater level of clarity than has been accomplished so far through examination of population-level responses. A positive response by a subset of individuals in a population may have implications for species genotype profiles and resilience globally.

Here, we looked at the relative responses to OA in two representative broadcast spawning invertebrates, the sea urchins *Lytechinus pictus* and *Heliocidaris erythrogramma*, that have different fertilization ecologies, where selection acting on sperm traits may therefore differ. As typical of most sea urchins, *Lytechinus pictus* has small sperm with a conical head (approx. 3.6 µm length, 1.3 µm diameter), small (approx. 100 µm diameter), negatively buoyant eggs and a feeding larva [32,33]. By contrast, *Heliocidaris erythrogramma* has large sperm with an elongated head (approx. 10.0 µm length, 2.0 µm diameter), large (approx. 400 µm diameter) positively buoyant eggs and a non-feeding larva [33,34]. Sea urchins are well-established model organisms for investigation of fertilization processes and the impacts of anthropogenic stressors [31]. We determined the level of variability of individual male reproductive performance (individual ejaculate traits and fertilization success) in response to OA conditions under non-competitive scenarios. Understanding how OA affects sperm function and fertilization success on an individual basis is required to build a better understanding of the potential that existing phenotypic variation in sperm performance traits will influence how marine species will respond globally.

## 2. Material and methods

### (a) Gamete collection and seawater manipulation

*Lytechinus pictus* were provided by Dunmanus Seafoods Ltd, Ireland, and *Heliocidaris erythrogramma* were collected from Sydney Harbour, Australia (see electronic supplementary material for details on animal collection). Gametes were obtained from both species using standard methods of injecting 0.5 mol KCl through the peristome [35]. Sperm were collected separately from each male ‘dry' using a pipette and maintained on ice prior to use. Eggs were collected in filtered seawater FSW (*L. pictus*, 15°C, salinity 35; *H. erythrogramma*, 20°C, salinity 35). Temperatures reflected the ambient temperature at collection for each species and were maintained for all subsequent work. In total, sperm was collected from 20 male *L. pictus* and 16 male *H. erythrogramma.* Eggs were collected from 10 females from each species. All experiments were conducted directly after spawning at the maintenance temperature ± 1°C. Experimental seawater was either aerated to simulate current ocean conditions (mean ± s.e., pH_NBS_ 8.07 ± 0.02, 506 ± 39 µatm *p*CO_2_), or received an input of CO_2_ pre-mixed with ambient air to achieve OA conditions (mean ± s.e., pH_NBS_ 7.90 ± 0.02, 792 ± 20 µatm *p*CO_2_ and pH_NBS_ 7.75 ± 0.01, 1170 ± 42 µatm *p*CO_2_). The CO_2_-air pre-mix was added via mass-flow controlled systems. Seawater samples were collected prior to experiments for assessment of carbonate chemistry (electronic supplementary material, table S1).

### (b) Sperm functional analysis

Sperm functional characteristics were analysed by computer-assisted sperm analysis (CASA) using Microptic Sperm Class Analyser and using ImageJ software [36] with a CASA plugin [37] (electronic supplementary material). Three subsets of sperm were examined from each individual 10 min after activation in seawater. A minimum of 250 individual sperm were tracked per male for 0.5 s during analysis. Measurements of sperm concentration, proportion of motile sperm (MOT) and six CASA parameters were used for further analysis. The CASA parameters included three measures of sperm velocity (curvilinear velocity [VCL], straight-line velocity [VSL] and average-path velocity [VAP]), two measures of sperm path (linearity [LIN; VSL/VCL] and straightness [STR; VSL/VAP]), and a measure of side-to-side sperm head movement (WOB; VAP/VCL). Immotile sperm were defined as sperm swimming below threshold values of 10 µm s^−1^ VCL and 3.2 µm s^−1^ VAP [38]. The data for each CASA parameter were further split by quartiles (low = 0–25th, medium = 25–75th and high = 75–100th) and corrected for motility to determine the distribution of total sperm across these categories for each male. For every CASA parameter, the percentiles were determined for each species using the data from all males under current conditions.

### (c) Fertilization success

Fertilizations were conducted using sperm from individual males and eggs pooled from 10 females. The individual male-pooled female design provided a ‘common' fertilization environment for the males thereby reducing variability caused by influential gamete incompatibility and increasing the likelihood of accurately identifying inter-individual trait variability in male responses to OA [39–40]. We note that this design does not consider the competitive fertilization scenario that can influence individual reproductive performance [24,25].

The eggs and sperm of *H. erythrogramma* are both considerably larger than those of *L. pictus.* Consequently, we used different oocyte-sperm concentrations for our fertilizations, selected to avoid either polyspermy or sperm limitation and achieve an average fertilization success rate of 75–80% under current conditions (10 000 eggs and a sperm concentration of 1 × 10^5^ for *L. pictus* [28] and 350 eggs and a sperm concentration of 1 × 10^4^ for *H. erythrogramma* [29]). Sperm from a single male was activated in the seawater treatment, mixed, then immediately pipetted into the centre of each well and gently mixed in a Z motion (*n* = 3 wells per male). Following controlled fertilizations where sperm were given 10 min to fertilize eggs, 100 eggs from each well were examined and the proportion of fertilized eggs determined. See electronic supplementary material for further details.

### (d) Statistical analysis and modelling

Nested ANOVAs (analysis of variance) were used to compare fertilization success and sperm functional parameters across conditions and across males within each condition (parameter ∼ condition (male)). Where necessary, data were transformed prior to analysis to ensure homoscedasticity. Sperm functional parameters from each male were also compared to fertilization success under each condition using Pearson's correlation. For *L. pictus*, 20 males were included in all analyses. For *H. erythrogramma*, 16 males were included in fertilization success analyses but only 14 males were included in sperm parameter analyses due to problems with CASA video analysis under current conditions. For multiple independent tests, Bonferroni correction was used to maintain an overall type I error rate, *α*, of 0.05.

The influence of sperm parameters on fertilization success under current conditions and separately under OA were explored using linear models. Models were built using the following basic structure:

$$Y=X_{1}+X_{2}+\ldots +X_{n}.$$

Twenty-six sperm functional parameters were included in different combinations (electronic supplementary material). Sperm parameters were added, substituted and removed to obtain the lowest Akaike information criterion (AIC) score. AIC values within 2.0 of each other were considered to be of equivalent statistical power. Beyond this, models were further optimized using the highest adjusted *R*^2^ and the highest number of degrees of freedom. Once the best-fitting model had been selected the model residuals were confirmed. Individual data points sitting outside 0.5 Cook's distance were examined and removed if deemed highly influential.

All statistical analyses were conducted in RStudio v. 1.1.423 [41] using the package ‘stats'. Figures were produced using GraphPad Prism v. 7.03 for Windows (GraphPad Software, La Jolla, CA, USA, www.graphpad.com).

## 3. Results

### (a) Sperm functional analysis

**Figure 1.**
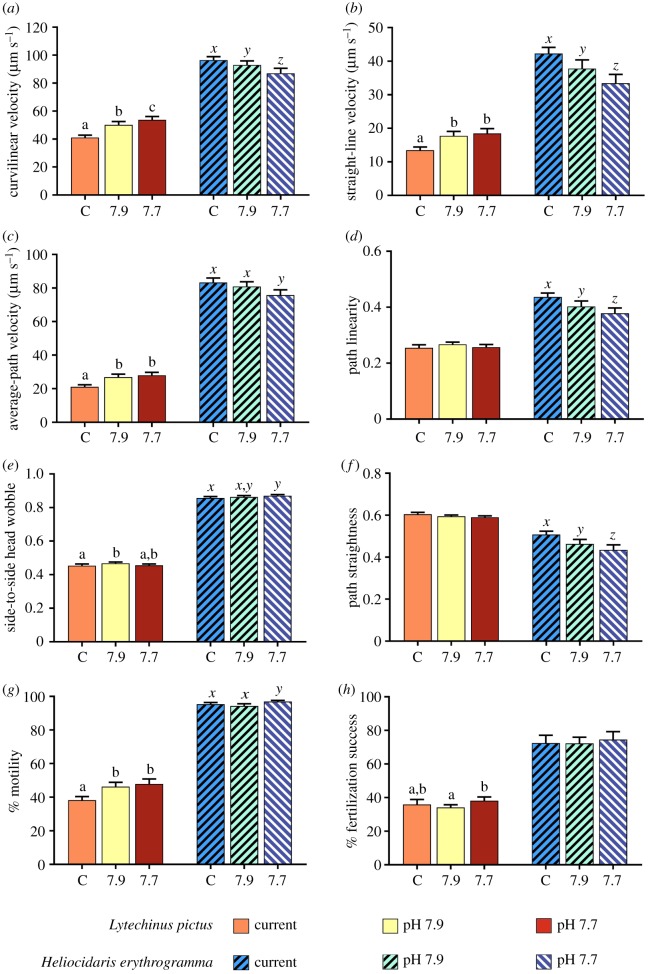
(*a–g*) Ejaculate traits and (*h*) fertilization success in *Lytechinus pictus* and *Heliocidaris erythrogramma*. Different letters above each trio of bars indicate a significant difference between treatments (*p* ≤ 0.05). (Online version in colour.)

When looking at average group responses for each species, *L. pictus* sperm increased significantly in velocity between current and OA conditions (VCL, from 40.50 to 52.95 µm s^−1^; VSL, from 13.12 to 18.00 µm s^−1^; VAP, from 20.60 to 27.51 µm s^−1^) but *H. erythrogramma* sperm instead decreased significantly in velocity (VCL, from 95.35 to 86.46 µm s^−1^; VSL, from 41.79 to 33.30 µm s^−1^; VAP, from 82.04 to 75.22 µm s^−1^; table 1 and figure 1*a–c*). OA did not affect average sperm LIN, WOB, or STR in *L. pictus*. There was also no impact of OA on sperm WOB in *H. erythrogramma*, but there was a significant decrease in both LIN (0.44 to 0.38) and STR (0.51 to 0.43) with increasing OA (table 1 and figure 1*d–f*). MOT was significantly affected by OA in both species (table 1 and figure 1*g*); an increase in sperm MOT in response to OA conditions was observed in *L. pictus* (from 38.24% under current conditions to 47.88% at pH 7.70) whereas in *H. erythrogramma*, MOT decreased at pH 7.90 but then increased slightly again at pH 7.70 (95.39% under current conditions, 94.28% at pH 7.90, 96.95% at pH 7.70). For summary details of all sperm functional parameters, see electronic supplementary material, table S2.

**Table 1.** Results of nested analysis of variance (sperm parameter ∼ condition (male)) examining sperm parameters in two species of echinoderm. The adjusted *α* following Bonferroni corrections was *p* = 0.007 for all tests. Significant *p-*values below the adjusted *α* are in italics.

species	*Lytechinus pictus*						*Heliocidaris erythrogramma*					
response variable	condition			condition (male)			condition			condition (male)		
sperm parameter	*F*	d.f.	*p*-value	*F*	d.f.	*p*-value	*F*	d.f.	*p*-value	*F*	d.f.	*p*-value
curvilinear velocity	101.56	2	*<0.001*	11.15	57	*<0.001*	35.60	2	*<0.001*	13.63	39	*<0.001*
straight-line velocity	67.49	2	*<0.001*	14.14	57	*<0.001*	62.67	2	*<0.001*	17.88	39	*<0.001*
average-path velocity	86.56	2	*<0.001*	14.18	57	*<0.001*	21.15	2	*<0.001*	11.11	39	*<0.001*
path linearity	2.76	2	0.067	3.42	57	*<0.001*	32.01	2	*<0.001*	11.81	39	*<0.001*
side-to-side head wobble	4.27	2	0.016	4.11	57	*<0.001*	3.84	2	0.025	4.61	39	*<0.001*
path straightness	1.07	2	0.346	1.59	57	0.018	39.76	2	*<0.001*	12.87	39	*<0.001*
motility	41.96	2	*<0.001*	7.92	57	*<0.001*	7.59	2	*<0.001*	5.34	39	*<0.001*

**Figure 2.**
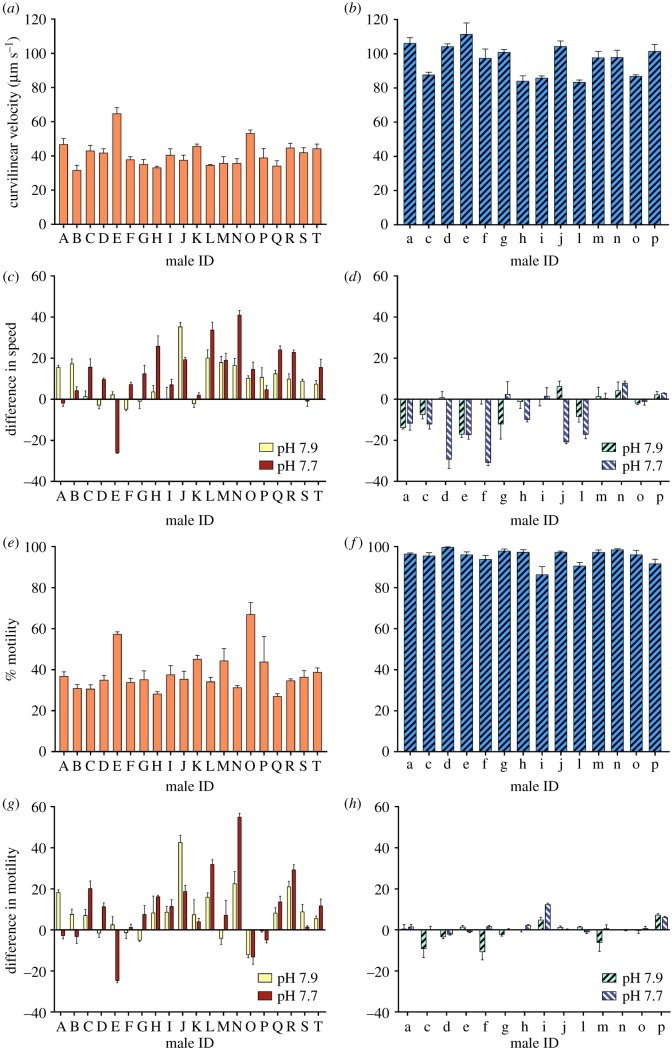
Ejaculate traits of *Lytechinus pictus* and *Heliocidaris erythrogramma*. (*a,b*) Sperm curvilinear velocity under current conditions; (*c,d*) difference in curvilinear velocity between current and OA conditions; (*e,f*) sperm motility under current conditions; (*g,h*) difference in motility between current and OA conditions. Different letters below each plot indicate ejaculate from individual males. (Online version in colour.)

In both species, the average VCL, VSL, VAP, LIN, WOB and MOT within the ejaculate for each individual differed significantly between males within each condition. Sperm STR also differed significantly between males within each condition in *H. erythrogramma* but not in *L. pictus* (table 1). Within a condition, variability between males increased with increasing OA in VCL, VSL and VAP in both species, in MOT in *L. pictus* and in LIN and STR in *H. erythrogramma.* Variability in LIN, WOB and STR between males decreased with increasing OA in *L. pictus.* In *H. erythrogramma* variability in MOT increased between current conditions and pH 7.90, before decreasing to pH 7.70. Variability in WOB remained similar across condition in this species (electronic supplementary material, table S2). Under current conditions, average ejaculate VCL ranged from 31.8 to 64.9 µm s^−1^ across all individual *L. pictus* (figure 2*a*). Under OA conditions, VCL changed by between −13.20 and +93.90% at pH 7.90 (per individual relative to current conditions; average 23.80% increase) and between −40.20 and +114.70% at pH 7.70 (average 34.80) across individuals (figure 2*c*). In *H. erythrogramma*, VCL ranged from 83.00 to 107.39 µm s^−1^ (average 96.09 µm s^−1^) across all individuals under current conditions (figure 2*b*). Under OA conditions, VCL changed by between −14.42 and +7.28% at pH 7.90 (average −3.60%) and by between −31.23 and +8.77 at pH 7.70 (average −9.78%) across individuals (figure 2*d*). Under current conditions, MOT ranged from 27.1 to 67.0% (average 38.2%) in *L. pictus* (figure 2*e*) and from 87.33 to 99.86% (average 95.31%) in *H. erythrogramma* (figure 2*f*). Relative to current conditions, MOT changed by between −18.30 and +120.70% across individuals at pH 7.90 (average 24.80%) and by between −43.30 and +175.80% at pH 7.70 (average 31.80%) in *L. pictus* (figure 2*g*). In *H. erythrogramma*, MOT changed by between −12.28 and +8.57% across individuals at pH 7.90 (average −1.19%) and by between −2.20 and +13.23% at pH 7.70 (average 1.77%) relative to current conditions (figure 2*h*).

### (b) Fertilization success

**Figure 3.**
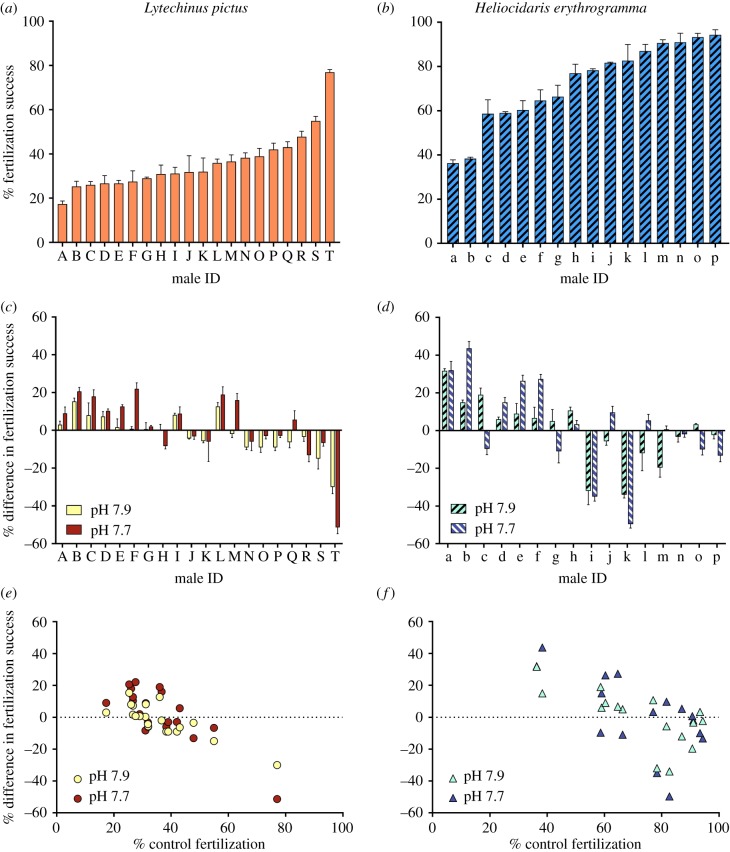
Fertilization success under different experimental conditions. (*a,b*) Fertilization success under current conditions, (*c,d*) difference in fertilization success between current and acidified conditions and (*e,f*) relationship between current fertilization and difference in fertilization under acidified conditions in *Lytechinus pictus* and *Heliocidaris erythrogramma*. Different letters below plots (*a*–*d*) indicate individual males. (Online version in colour.)

When looking at average group responses for each species, in *L. pictus* there was a small but significant change in fertilization success with OA (ANOVA: *F* = 5.73, d.f. = 2, *p* = 0.004). Average fertilization success decreased significantly between current conditions and pH 7.90 (from 35.97% to 34.18%), and then increased again at pH 7.70 (38.15%). Fertilization success at pH 7.70 was not significantly different to either of the other treatments (figure 1*h*). By contrast, OA did not affect average fertilization success in *H. erythrogramma* (ANOVA: *F* = 1.85, d.f. = 2, *p* = 0.164). However, for each species and within each condition, fertilization success varied significantly between males (ANOVA: *L. pictus*, *F* = 8.12, d.f. = 57, *p* < 0.001; *H. erythrogramma*, *F* = 15.83, d.f. = 39, *p* < 0.001). Under current conditions, fertilization success ranged from 17.33% to 77.00% in *L. pictus* (figure 3*a*) and from 36.33% to 94.33% in *H. erythrogramma* (figure 3*b*). Under OA, fertilization success changed by between −30.00% and +15.33% at pH 7.90 and between −51.33% and +22.05% at pH 7.70 across individuals in *L. pictus* (figure 3*c*). In *H. erythrogramma*, fertilization success changed by between −34.00% and +31.67% at pH 7.90 and between −49.67% and +43.67% at pH 7.70 (figure 3*d*). The lower-than-expected fertilization success in *L. pictus* was probably because this species has smaller eggs and sperm than *Mesocentrotus franciscanus*, on which the experimental ratios were based [28].

In both species, males with low fertilization success under current conditions showed improved fertilization under OA, whereas the males with high fertilization success under current conditions worsened under OA (figure 3*a–d*). Spearman correlation revealed a strong relationship between fertilization under current conditions and the change in fertilization under OA for both *L. pictus* (pH 7.9: *r_s_* = −0.809, d.f. = 18, *p* < 0.001; pH 7.7: *r_s_* = −0.656, d.f. = 18, *p* < 0.001; figure 3*e*) and *H. erythrogramma* (pH 7.9: *r_s_* = −0.741, d.f. = 14, *p* < 0.001; pH 7.7: *r_s_* = −0.618, d.f. = 14, *p* = 0.006; figure 3*f*). The changes in fertilization with OA led to an overall shift in individual rankings with regard to fertilization success (electronic supplementary material, figure S1). For each species, the observed changes in fertilization success typically scaled with increasing pH: individuals that improved under pH 7.9 improved further under pH 7.7, and vice versa for those that worsened (figure 3*c,d*). In *L. pictus*, the change in fertilization success relative to current fertilization ranged from +60.53% to −38.96% at pH 7.9 and +81.58% to −66.67% at pH 7.7. In *H. erythrogramma,* the observed changes ranged from +87.16% to −41.13% at pH 7.9 and +113.91% to −60.08% at pH 7.7. See electronic supplementary material, table S2 for summary data.

### (c) Sperm functional analysis and fertilization success

**Figure 4.**
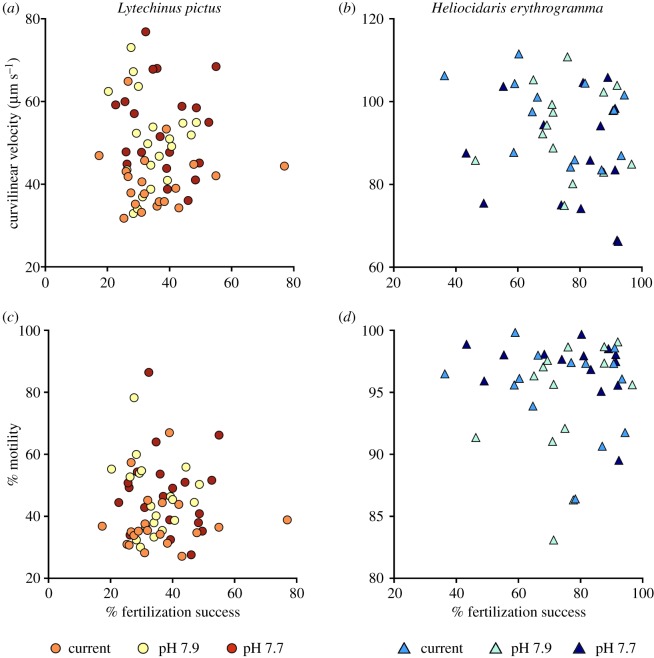
Relationship between fertilization success and key sperm parameters. (*a,b*) Fertilization success and curvilinear velocity, and (*c,d*) fertilization success and motility in *Lytechinus pictus* and *Heliocidaris erythrogramma*. (Online version in colour.)

Pearson's correlation indicated no relationship between fertilization success and any measurement of sperm function (VCL, VSL, VAP, LIN, WOB, STR, MOT) for either species under current conditions, following Bonferroni adjustments (electronic supplementary material, table S3). There were also no relationships observed under OA conditions. The relationships between fertilization success and VCL, and fertilization success and MOT are displayed in figure 4.

The sperm functional parameters contributing to the ‘best-fitting' linear model explaining fertilization success under current conditions varied between the two species. For *L. pictus*, the ‘best-fitting' LM explained 76.97% of the observed variation in fertilization success under current conditions (*F* = 8.94, d.f. = 11, _adj_*R*^2^ = 0.7697, *p* < 0.001, AIC = 79.32). Based on the resulting *β* values, the most important sperm functional parameters for predicting fertilization success were low-to-medium swimming speed (VCL and VAP), medium path linearity and side-to-side head wobble, and low path straightness. For *H. erythrogramma,* the ‘best-fitting' LM explained 93.38% of the observed variation in fertilization success under current conditions (*F* = 31.56, d.f. = 7, _adj_*R*^2^ = 0.9338, *p* < 0.001, AIC = 45.85). Based on the resulting *β* values, the most important sperm functional parameters for predicting fertilization success in *H. erythrogramma* were fast swimming speed (VCL) and a straight path (see electronic supplementary material, table S4 for model terms, *p* and *β* values).

Under OA conditions, the sperm functional parameters contributing to the ‘best-fitting' LM explaining fertilization success shifted for both species. For *L. pictus*, the ‘best-fitting' LM explained 66.20% of the fertilization success under experimental conditions (*F* = 5.830, d.f. = 22, _adj_*R^2^* = 0.662, *p* < 0.001, AIC = 134.94). Based on the resulting *β* values, the most important sperm functional parameters for predicting fertilization success in *L. pictus* under OA were fast swimming speed, low path straightness and medium-to-high side-to-side head wobble. For *H. erythrogramma*, the ‘best-fitting' LM explained 84.52% of the change in fertilization success between current and experimental conditions (*F* = 11.50, d.f. = 12, _adj_*R*^2^ = 0.845, *p* < 0.001, AIC = 100.89). Based on the resulting *β* values, the most important sperm functional parameters for predicting fertilization success under OA conditions in *H. erythrogramma* were medium-to-high swimming speed and low path linearity (see electronic supplementary material, table S5 for model terms, *p* and *β* values).

## 4. Discussion

OA is predicted to be a significant driver of global ocean biodiversity change this century, with species-specific ‘winners' and ‘losers' already being estimated based on population-level sensitivities [1,42]. Our data clearly illustrate that under non-competitive scenarios, within a species both increases and decreases in male performance can occur based on the response of sperm within a single ejaculate, with some individuals performing better under OA conditions. Importantly, we found an inverse relationship between male reproductive performance (measured as fertilization success) under current ocean conditions and change in reproductive performance under future ocean conditions. The males who performed the best under current conditions typically worsened under OA, while those who were the least successful under current conditions generally improved under OA. The same trend was evident in both *L. pictus* and *H. erythrogramma* despite the differences in their fertilization ecology. Interestingly, although to our knowledge this relationship has not previously been described, we re-examined data from the only comparable study we could find that reported data for individual males fertilized with multiple females [30] (electronic supplementary material, table S6). We identified a similar inverse relationship for males under OA in *Crassostrea gigas*, where following initial non-competitive fertilization success ranging from 29% to 92%, fertilization success changed by between −11% and +9% under OA (pH 7.80) conditions (electronic supplementary material, figure S2) [30].

Collectively these observed trends in fertilization success indicate the optimum pH for sperm performance varies on a species and individual male level. The differing sperm responses to pH and pCO_2_ may be linked to a number of biochemical and/or physiological mechanisms. For example, activation of sperm swimming involves numerous enzymes all of which have optimal pH values [43] that may differ between both individuals and species. Sperm nuclear proteins, the differing ratios of which are known to influence sperm shape and motility and hence fertility [26], may also vary between individuals. Additionally, sperm traits can be modified by epigenetic mechanisms during their development which can also influence sperm performance [22].

The relationship between average sperm swimming performance and fertilization success has been relatively well explored for broadcast spawners [24,25,35,44], but it has rarely been examined in response to OA (but see [28,29]). A change in sperm swimming performance may alter its oocyte searching efficiency, directly influencing fertilization rates. Sperm parameters including motility, velocity, length and longevity have previously been identified to partially influence fertilization success in echinoderms and other phyla [15,27,35,39,44], but we found no relationship between any single sperm functional parameter and fertilization success under the non-competitive (single male) scenarios studied here. Instead, we found that fertilization was best predicted by a range of sperm parameters that varied between both species and conditions. Under current conditions, low-to-medium speeds and low path linearity were important for predicting fertilization success in *L. pictus* whereas high speed and high path linearity were important in *H. erythrogramma*. Under OA conditions, a straighter, faster sperm path was more important for predicting fertilization success in *L. pictus.* Conversely, a slower, less linear path was more important in *H. erythrogramma.* The shifts in important parameters mirrored the observed changes in sperm functional parameters; sperm swimming speeds typically increased in *L. pictus* individuals in response to increasing OA but decreased in *H. erythrogramma*. Similar contrasting shifts in sperm parameters have been reported for two species of *Helicidaris* in response to OA [34], decreases in sperm velocity and path straightness were observed in *H. erythrogramma,* whereas in *H. tuberculata*, which has similar life-history mode to *L. pictus* (small eggs and planktotrophic larvae), sperm velocity and path linearity increased.

The observed differences in the most beneficial sperm functional traits for fertilization under current conditions between the two species studied here may be partially explained by their different reproductive ecologies. Fast swimming speeds are likely to be most beneficial for *H. erythrogramma* sperm which must actively swim upwards through the water column to reach the large, buoyant eggs of this species. Conversely, slower speeds may be more evolutionarily beneficial for *L. pictus* if they enable sperm longer periods to search for their small eggs [20,35,39]. Considering this, OA appears to reduce the influence of the most beneficial sperm functional trait for fertilization in both species (i.e. by increasing swimming speeds in *L. pictus* but reducing them in *H. erythrogramma*). Fertilization success is also influenced by other factors including sperm chemotaxis and egg penetration. For both species it is likely that some sperm were placed closer to an egg than others due to the mixing action and may have resulted in some egg–sperm contact independent of sperm swimming parameters. However, mixing is standard in fertilization studies and the methods were the same for each species.

The individual variability in sperm activity and fertilization success observed here is not surprising; inter-individual variability in sperm traits is an inherent feature of male ejaculate in broadcast spawning marine invertebrates [6,7,27,31,35]. Thus, the variability of sperm responses under OA integrates with a standing phenotypic variation and has been reported several times in response to OA [5,28–30]. Consequently, inherent variability may also help explain the conflicting data reported in previous studies examining population-level responses to OA [7–11,31]. The results of these studies are often interpreted to indicate population-level losses or gains under future ocean conditions, depending on whether the observed response is negative or positive. Our results instead highlight the importance of recognizing inter-individual variability; while the ‘average' impact of OA clearly varies species by species, individual variability within a species may drive selection, indicating a greater level of species resilience than is often interpreted. It is important to note that our experimental focus was to determine outcomes for individual males and that their performance might differ under multiple male competitive fertilization scenarios.

The variability in sperm traits identified across individuals in the present study is likely to be related to environmental heterogeneity; both *L. pictus* and *H. erythrogramma* inhabit coastal habitats where environmental variability is high and can change significantly over short spatial and temporal periods [45,46]. Individuals that are adapted to higher levels of environmental variability exhibit greater plasticity and physiological tolerances than those adapted to narrow variable regimes [47]. Environmental conditions both pre- and post-gamete release can impact sperm phenotype, with different sperm characteristics favoured under different conditions (for review see [22]). The degree to which individuals are affected may be related to genotype-by-environment interactions although further analysis is required to determine this [26,45,48,49]. Individuals and populations of the same species can also exhibit varying responses to an environmental stressor (e.g. OA, temperature, salinity), and compounding or alleviating effects of multiple stressors vary case-by-case [15,48,50]. By increasing variability in sperm traits, individuals are more likely to have some sperm that fertilize successfully across the environmental conditions they are exposed to.

Understanding individual variability within populations is clearly of great importance for predicting species resilience. A vast proportion of marine invertebrates reproduce via broadcast spawning, including many ecologically and economically important species. Interpreting how OA will impact these species is of utmost importance, and it is clear from our results that key trends in individual resilience may not be identified using a population-level approach. If the sperm functional traits that are important for fertilization success are heritable for *H. erythrogramma* and *L. pictus*, and the competitiveness of individual males also shifts with OA (as shown previously [28]), the contributions by each male to the population might shift as their fertilization capacity changes. However, the overall population numbers may not change significantly. It remains to be seen whether the same trend is evident across other species. Our results highlight the importance of examining responses to environmental change at an individual level when interpreting how future ocean conditions will affect populations of marine invertebrates; such knowledge is fundamental for predicting biodiversity change across the shifting OA landscape globally.

## Supplementary Material

Supplementary Information

Reviewer comments

## Data accessibility

The datasets supporting this article have been uploaded as part of the electronic supplementary material.
