# Supplementary material for: Sea urchin reproductive performance in a changing ocean: poor males improve while good males worsen in response to ocean acidification
Source: Proc Biol Sci. 2019 Jul 24;286(1907):20190785. doi: 10.1098/rspb.2019.0785 (PMC6661356; doi:10.1098/rspb.2019.0785)
Supplement: Supplementary Information [file rspb20190785supp1.docx]

**Smith et al. 2018 Supplementary information**

**Sea urchin reproductive performance in a changing ocean: poor males improve while good males worsen in response to ocean acidification**

Kathryn E. Smith, Maria Byrne, Dione Deaker, Cameron M. Hird, Clara Nielson, Alice Wilson-McNeal, Ceri Lewis.

**Collection of animals**

*Lytechinus pictus* (test diameter 31.35 ± 0.66, mean ± S.E.), native to California, were purchased from Dunmanus Seafood Ltd, Co. Cork, Ireland, in March 2017, transported to the Aquatic Research Centre at the University of Exeter (UoE), UK, and maintained together in artificial filtered seawater until use (ASW, 0.2 µm filtered, 15°C, salinity 34, 400 µatm *p*CO_2_). They were fed *ad libitum* on a diet of fresh *Laminaria saccharina. Heliocidaris erythrogramma* (test diameter 53.04 ± 1.45, mean ± S.E.) were collected by hand from Milk Beach, Sydney Harbour, Australia (33°51′23″S, 151°16′11″E) in November 2017 and transported to the Sydney Institute of Marine Science (SIMS). These urchins were maintained in the aquarium system at SIMS until use, supplied by flow-through ambient filtered seawater (FSW, 1 µm, 20°C, salinity 35) from Sydney Harbour with intake at 8 m in Chowder Bay.

Seawater samples were collected from each experimental condition directly prior to use and fixed (0.04% of final volume) with 4% mercuric chloride as per [51]. Dissolved inorganic carbon (DIC) analysis was carried out on ASW samples in-house at the UoE whereas FSW samples were analysed for total alkalinity (TA) at the University of Sydney. DIC analysis was carried out using a bespoke system detailed in [52] and following methodology described by [53]. This system allowed seawater DIC to be measured with a precision of ± µM. TA was determined by potentiometric titration (907 Titrando, Metrohm), calibrated using sea water CRMs (Batch 139, [51]). Final values for DIC, TA and pCO2 were calculated using CO2SYS ([54], Supplementary Table 1) according to [55,56]. The pH of FSW was also confirmed on the day of the experiments by spectrophotometer at SIMS.

**Sperm functional analysis**

Sperm concentration was determined for each male by haemocytometer count following the serial dilution of 1 µl sperm. Sperm were activated in each seawater condition at a final concentration of 1 x 10^6^ and incubated for 10 minutes at the species maintenance temperature. This time period was used to maximise swimming performance and ensure sperm were fully activated but not aging^57^.

For *L. pictus,* activated sperm were analysed via a Microptic Sperm Class Analyser® (SCA: Microm, UK) fitted with a Nikon Eclipse 50i negative phase contrast microscope (100 x magnification) and a Peltier cooled stage set to 15°C. For *H. erythrogramma*, activated sperm were transferred to a microscope slide and recorded for 2-seconds with an image capture rate of 29 frames/second (Olympus DP73 digital camera mounted on an Olympus BX60 microscope), inside a temperature-controlled room (20°C). Slides and cover slips were prepared using a 1 % bovine serum albumin solution to prevent sperm from sticking to the glass. Cover slips were suspended ~ 2 mm above the slide using plasticine supports. Each video was analysed *post hoc* using Image J software^36^ with a CASA plugin^37^.

**Fertilisation success**

Fertilisations were carried out in 6-well plates, each containing 10 ml of treatment water. The ratio of oocytes to sperm for each species was based on previous studies ([28] and [29] for *L. pictus* and *H. erythrogramma*, respectively). Either 10,000 *L. pictus* oocytes or 350 *H. erythrogramma* oocytes were added to each well, equally contributed to by 10 females from each species. After 10 minutes, the water was gently pipetted out of each well and replaced with fresh treatment water to remove sperm and prevent subsequent fertilisations. This step was repeated twice in quick succession. Prior to carrying out fertilisations, sperm activity was tested over an hour to confirm sperm longevity was as is typical of sea urchins^35^ and significantly longer than the experimental period we were using sperm over. All fertilisations took place at the maintenance temperature for each species. The fertilisation plates were maintained at this temperature overnight before being fixed with Paraformaldehyde (4 %) to prevent further development. One hundred oocytes from each well were then examined under the microscope for fertilisation success.

**Model parameters**

Twenty-six sperm functional parameters were included in the linear modelling optimisation. These included average CASA parameters and the proportion of total sperm in each parameter that fell into the low/medium/high quartile split.

The complete list of parameters investigated was:

VCL (average)

VSL (average)

VAP (average)

LIN (average)

STR (average)

WOB (average)

VCL (low)

VCL (medium)

VCL (high)

VSL (low)

VSL (medium)

VSL (high)

VAP (low)

VAP (medium)

VAP (high)

LIN (low)

LIN (medium)

LIN (high)

STR (low)

STR (medium)

STR (high)

WOB (low)

WOB (medium)

WOB (high)

MOT

Sperm conc.

**References:**

28. Campbell, A. L., Levitan, D. R., Hosken, D. J., & Lewis, C. (2016). Ocean acidification changes the male fitness landscape. *Scientific reports*, *6*, 31250.

29. Schlegel, P., Havenhand, J. N., Gillings, M. R., & Williamson, J. E. (2012). Individual variability in reproductive success determines winners and losers under ocean acidification: a case study with sea urchins. *PloS one*, *7*(12), e53118.

30. Havenhand, J. N., & Schlegel, P. (2009). Near-future levels of ocean acidification do not affect sperm motility and fertilization kinetics in the oyster *Crassostrea gigas*. *Biogeosciences*, *6*(12), 3009–3015.

35. Levitan DR. 2000. Sperm velocity and longevity trade off each other and influence fertilization in the sea urchin *Lytechinus variegatus*. *Proc. R. Soc. B*, 267(1443), 531–534.

36. Schneider, C.A., Rasband, W.S., & Eliceiri, K.W. (2012), "[NIH Image to ImageJ: 25 years of image analysis](http://www.nature.com/nmeth/journal/v9/n7/full/nmeth.2089.html)", Nature methods 9(7): 671–675.

37. Wilson-Leedy, J.G., & Ingermann, R.L. (2006). Development of a novel CASA system based on open source software for characterization of zebrafish sperm motility parameters. *Theriogenology*. *67*(3): 661–672.

51. Dickson, A., Sabine, C., Christian, J. (2007) Guide to Best Practices for Ocean CO_2_ measurements.

52. Friederich, G., Walz, P., Burczynski, M., Chavez, F. (2002) Inorganic carbon in the central California upwelling system during the 1997–1999 El Niño–La Niña event. *Progress in Oceanography*. 54, 185–203.

53. Lewis, C., Clemow, K., Holt, W.V. (2012) Metal contamination increases the sensitivity of larvae but not gametes to ocean acidification in the polychaete *Pomatoceros lamarckii* (Quatrefaces). *Marine Biology*. 160, 2089–2101.

54. Pierrot, D., Lewis, E., Wallace, D. (2006) MS excel program developed for CO_2_ system calculation. ORNL/CDIAC-105a. Carbon Dioxide Information Analysis Center, Oak Ridge National Laboratory, US Department of Energy, Oak Ridge, Tennessee.

55. Campbell, A.L., Ellis, R.P., Urbina, M.A., Mourabit, S., Galloway, T.S., Lewis, C. (2017). Impacts of ocean acidification on sperm develop with exposure time for a polychaete with long lived sperm. *Marine Environmental Research.* 129, 268–276.

56. Findlay, H.S., Artioli, Y., Moreno Navas, J., Hennige, S.J., Wicks, L.C., Huvenne, V.A., Woodward, E.M.S., Roberts, J.M. (2013) Tidal downwelling and implications for the carbon biogeochemistry of cold-water corals in relation to future ocean acidification and warming. *Global Change Biology*. 19, 2708–2719.

57. Mortimer, D., Aitken, R.J., Mortimer, S.T., & Pacey, A.A. (1995). Workshop report: clinical CASA--the quest for consensus. *Reproduction, Fertility and Development*, *7*(4), 951–959.

**Supplementary Table 1: Carbonate chemistry (measured and calculated using CO2SYS^51^).** Seawater parameters for water used in experimental treatments (n = 2 per treatment). Data displayed as averages ± SE. NOTE: the ambient in situ pH range for the population of *Heliocidaris erythrogramma* studied here is approximately pH 7.95 to almost pH 8.15 (Byrne, unpublished data). There are no data available for the pH range in the *Lytechinus pictus* habitat.

| **Treatment** | **Temp (°C)** | **Salinity** | **pH_NBS_** | **DIC (µmol kg^-1^)** | **TA (µmol kg^-1^)** | ***p*CO_2_ (µatm)** | **HCO_3_^-^ (µmol kg^-1^)** | **CO_3_^2-^ (µmol kg^-1^)** |
| --- | --- | --- | --- | --- | --- | --- | --- | --- |
| **Control (UK)** | **15.01 ± 0.01** | **33.63 ± 0.05** | **8.11 ± 0.01** | **1920 ± 0.5** | **2096 ± 3.95** | **423 ± 10** | **1778 ± 2.14** | **127 ± 2.47** |
| **pH 7.9 (UK)** | **15.10 ± 0.01** | **33.60 ± 0.06** | **7.88 ± 0.01** | **2017 ± 0.75** | **2109 ± 3.65** | **771 ± 26** | **1910 ± 1.08** | **81 ± 1.91** |
| **pH 7.7 (UK)** | **15.12 ± 0.01** | **33.57 ± 0.12** | **7.75 ± 0.01** | **2074 ± 1.25** | **2127 ± 2.65** | **1076 ± 26** | **1973 ± 0.40** | **61 ± 1.23** |
| **Control (Aus)** | **19.86 ± 0.02** | **34.80 ± 0.00** | **8.04 ± 0.00** | **2173 ± 0.26** | **2367 ± 0.27** | **589 ± 2** | **2006 ± 0.23** | **147 ± 0.02** |
| **pH 7.9 (Aus)** | **19.87 ± 0.01** | **34.80 ±0.00** | **7.94 ± 0.00** | **2204 ± 0.06** | **2353 ± 0.47** | **760 ± 4** | **2059 ± 0.31** | **120 ± 0.32** |
| **pH 7.7 (Aus)** | **19.95 ± 0.01** | **34.80 ± 0.00** | **7.73 ± 0.01** | **2281 ± 1.49** | **2352 ± 2.84** | **1295 ± 15** | **2161 ± 1.08** | **78 ± 0.74** |

**Supplementary Table 2: Summary data for sperm functional parameters and fertilisation success.**

|  | ***Species: Lytechinus pictus*** |  | | | |
| --- | --- | --- | --- | --- | --- |
|  |  | **Averages per male** | | | |
| **Condition** | **Sperm parameter** | **Max.** | **Min.** | **Mean** | **S.D.** |
| Current | Sperm curvilinear velocity (µm s^-1^) | 64.88 | 31.76 | 40.50 | 7.80 |
|  | Sperm straight-line velocity (µm s^-1^) | 26.59 | 7.95 | 13.12 | 4.35 |
|  | Sperm average-path velocity (µm s^-1^) | 36.95 | 14.80 | 20.60 | 5.63 |
|  | Sperm path linearity | 0.37 | 0.18 | 0.25 | 0.05 |
|  | Sperm side-to-side head wobble | 0.55 | 0.39 | 0.45 | 0.04 |
|  | Sperm path straightness | 0.71 | 0.55 | 0.61 | 0.04 |
|  | Sperm motility (%) | 67.00 | 27.13 | 36.73 | 9.66 |
|  | Fertilisation success (%) | 77.00 | 17.33 | 36.23 | 13.01 |
| pH 7.90 | Sperm curvilinear velocity (µm s^-1^) | 73.06 | 32.89 | 49.42 | 10.88 |
|  | Sperm straight-line velocity (µm s^-1^) | 29.03 | 8.71 | 17.29 | 5.69 |
|  | Sperm average-path velocity (µm s^-1^) | 43.93 | 15.10 | 26.45 | 7.53 |
|  | Sperm path linearity | 0.33 | 0.21 | 0.27 | 0.04 |
|  | Sperm side-to-side head wobble | 0.55 | 0.42 | 0.47 | 0.03 |
|  | Sperm path straightness | 0.64 | 0.561 | 0.59 | 0.03 |
|  | Sperm motility (%) | 78.30 | 30.08 | 45.84 | 11.66 |
|  | Fertilisation success (%) | 48.67 | 20.33 | 34.33 | 7.33 |
| pH 7.70 | Sperm curvilinear velocity (µm s^-1^) | 76.85 | 36.06 | 52.95 | 11.11 |
|  | Sperm straight-line velocity (µm s^-1^) | 27.60 | 8.07 | 18.00 | 6.26 |
|  | Sperm average-path velocity (µm s^-1^) | 41.27 | 15.55 | 27.51 | 7.63 |
|  | Sperm path linearity | 0.32 | 0.17 | 0.25 | 0.04 |
|  | Sperm side-to-side head wobble | 0.51 | 0.38 | 0.45 | 0.04 |
|  | Sperm path straightness | 0.64 | 0.54 | 0.59 | 0.03 |
|  | Sperm motility (%) | 86.43 | 27.62 | 47.57 | 13.49 |
|  | Fertilisation success (%) | 55.00 | 22.67 | 38.97 | 9.78 |
|  | ***Species: Heliocidaris erythrogramma*** | | | | |
|  |  | **Averages per male** | | | |
| **Condition** | **Sperm parameter** | **Max** | **Min** | **Mean** | **S.D.** |
| Current | Sperm curvilinear velocity (µm s^-1^) | 111.52 | 83.51 | 95.35 | 9.19 |
|  | Sperm straight-line velocity (µm s^-1^) | 53.06 | 28.55 | 41.79 | 6.96 |
|  | Sperm average-path velocity (µm s^-1^) | 99.65 | 67.25 | 82.04 | 9.80 |
|  | Sperm path linearity | 0.54 | 0.33 | 0.44 | 0.05 |
|  | Sperm side-to-side head wobble | 0.89 | 0.79 | 0.85 | 0.03 |
|  | Sperm path straightness | 0.62 | 0.37 | 0.51 | 0.06 |
|  | Sperm motility (%) | 99.84 | 86.41 | 95.34 | 3.61 |
|  | Fertilisation success (%) | 94.33 | 36.33 | 71.80 | 18.50 |
| pH 7.90 | Sperm curvilinear velocity (µm s^-1^) | 110.81 | 74.92 | 92.99 | 10.61 |
|  | Sperm straight-line velocity (µm s^-1^) | 56.09 | 23.64 | 37.56 | 9.68 |
|  | Sperm average-path velocity (µm s^-1^) | 101.65 | 65.29 | 81.10 | 10.12 |
|  | Sperm path linearity | 0.51 | 0.27 | 0.40 | 0.07 |
|  | Sperm side-to-side head wobble | 0.92 | 0.82 | 0.86 | 0.03 |
|  | Sperm path straightness | 0.55 | 0.31 | 0.46 | 0.08 |
|  | Sperm motility (%) | 99.07 | 83.09 | 94.03 | 4.88 |
|  | Fertilisation success (%) | 96.67 | 46.33 | 73.89 | 14.56 |
| pH 7.70 | Sperm curvilinear velocity (µm s^-1^) | 105.86 | 66.23 | 86.46 | 13.67 |
|  | Sperm straight-line velocity (µm s^-1^) | 49.19 | 20.93 | 33.30 | 9.82 |
|  | Sperm average-path velocity (µm s^-1^) | 90.55 | 57.05 | 75.22 | 11.84 |
|  | Sperm path linearity | 0.48 | 0.26 | 0.38 | 0.07 |
|  | Sperm side-to-side head wobble | 0.91 | 0.82 | 0.87 | 0.03 |
|  | Sperm path straightness | 0.58 | 0.28 | 0.44 | 0.09 |
|  | Sperm motility (%) | 99.68 | 89.52 | 97.09 | 2.49 |
|  | Fertilisation success (%) | 92.33 | 33.00 | 77.29 | 19.16 |

**Supplementary Table 3: Results of Pearson’s correlation comparing sperm parameters with fertilisation success. The adjusted α following Bonferroni corrections was *P* = 0.007 for all tests.** **Significant *p* values below the adjusted α are in bold.**

| ***Species: Lytechinus pictus*** | | | | |
| --- | --- | --- | --- | --- |
| **Condition** | **Sperm parameter** | **r** | **df** | ***P*** |
| Current | Curvilinear velocity | 0.002 | 18 | 0.992 |
|  | Straight-line velocity | -0.027 | 18 | 0.912 |
|  | Average-path velocity | 0.027 | 18 | 0.910 |
|  | Path linearity | -0.287 | 18 | 0.219 |
|  | Side-to-side head wobble | 0.137 | 18 | 0.564 |
|  | Path straightness | -0.320 | 18 | 0.169 |
|  | Motility | 0.044 | 18 | 0.853 |
| pH 7.90 | Curvilinear velocity | -0.011 | 18 | 0.964 |
|  | Straight-line velocity | -0.028 | 18 | 0.905 |
|  | Average-path velocity | -0.034 | 18 | 0.886 |
|  | Path linearity | 0.016 | 18 | 0.947 |
|  | Side-to-side head wobble | -0.037 | 18 | 0.879 |
|  | Path straightness | -0.136 | 18 | 0.568 |
|  | Motility | -0.127 | 18 | 0.593 |
| pH 7.70 | Curvilinear velocity | -0.104 | 18 | 0.663 |
|  | Straight-line velocity | -0.059 | 18 | 0.806 |
|  | Average-path velocity | -0.100 | 18 | 0.674 |
|  | Path linearity | -0.098 | 18 | 0.681 |
|  | Side-to-side head wobble | -0.125 | 18 | 0.600 |
|  | Path straightness | 0.147 | 18 | 0.537 |
|  | Motility | -0.097 | 18 | 0.685 |
|  |  |  |  |  |
| ***Species: Heliocidaris erythrogramma*** | | | | |
| **Response variable** | **Sperm parameter** | **r** | **df** | ***P*** |
| Current | Curvilinear velocity | -0.403 | 12 | 0.153 |
|  | Straight-line velocity | 0.153 | 12 | 0.6.3 |
|  | Average-path velocity | -0.381 | 12 | 0.179 |
|  | Path linearity | 0.514 | 12 | 0.060 |
|  | Side-to-side head wobble | -0.193 | 12 | 0.501 |
|  | Path straightness | 0.574 | 12 | 0.847 |
|  | Motility | -0.259 | 12 | 0.371 |
| pH 7.90 | Curvilinear velocity | 0.023 | 12 | 0.938 |
|  | Straight-line velocity | 0.377 | 12 | 0.184 |
|  | Average-path velocity | 0.064 | 12 | 0.829 |
|  | Path linearity | 0.540 | 12 | 0.046 |
|  | Side-to-side head wobble | 0.156 | 12 | 0.594 |
|  | Path straightness | 0.521 | 12 | 0.056 |
|  | Motility | 0.305 | 12 | 0.289 |
| pH 7.70 | Curvilinear velocity | -0.097 | 12 | 0.741 |
|  | Straight-line velocity | 0.104 | 12 | 0.724 |
|  | Average-path velocity | -0.196 | 12 | 0.503 |
|  | Path linearity | 0.204 | 12 | 0.203 |
|  | Side-to-side head wobble | -0.561 | 12 | 0.037 |
|  | Path straightness | 0.265 | 12 | 0.359 |
|  | Motility | -0.253 | 12 | 0.327 |

**Supplementary Table 4: Sperm functional terms contributing to the ‘best fitting’ models for predicting fertilisation success under ambient conditions. Significant *p* values (*p ≤* 0.05) are in bold.**

| ***Lytechinus pictus*** | | | | | | |
| --- | --- | --- | --- | --- | --- | --- |
| ***Model: lm(Fertilisation ~ LIN(med) + VAP(low) + VAP(med) + VCL(med) + STR(low) + STR(high) + LIN(avg) + WOB(avg))*** | | | | | | |
| **Coefficient** | ***p*** | **β** |  | **Coefficient** | ***p*** | **β** |
| Intercept | 0.773 | 0.000 |  | STR(low) | **< 0.001** | -2.517 |
| LIN(med) | **< 0.001** | -2.404 |  | STR(high) | **0.012** | -1.628 |
| VAP(low) | **< 0.001** | 2.083 |  | LIN(avg) | **0.020** | 4.294 |
| VAP(med) | **< 0.001** | 4.768 |  | WOB(avg) | 0.067 | -2.540 |
| VCL(med) | **0.006** | 2.209 |  |  |  |  |
| ***Heliocidaris erythrogramma*** | | | | | | |
| ***Model:lm(Fertilisation ~ LIN(low) + LIN(high) + VAP(med) + VCL(high) + ­­­WOB(med) + VCL(avg))*** | | | | | | |
| **Coefficient** | ***p*** | **β** |  | **Coefficient** | ***p*** | **β** |
| Intercept | **0.007** | 0.000 |  | *VCL(high)* | **0.031** | 2.917 |
| STR(low) | 0.057 | 0.391 |  | *WOB(med)* | **0.001** | -0.750 |
| *STR(high)* | **< 0.001** | 1.064 |  | *VCL(avg)* | **0.019** | 1.064 |
| *VAP(med)* | 0.137 | 0.593 |  |  |  |  |

VCL = curvilinear velocity of sperm path; VAP = average sperm path velocity; LIN = sperm path linearity; STR = sperm path straightness; WOB = side-to-side wobble of sperm head.

**Supplementary Table 5: Sperm functional terms contributing to the ‘best fitting’ models for predicting fertilisation success under future (OA) conditions. Significant *p* values (*p ≤* 0.05) are in bold.**

| ***Lytechinus pictus*** | | | | | | | |
| --- | --- | --- | --- | --- | --- | --- | --- |
| ***Model: lm(Fertilisation ~Sperm concentration + VCL(avg) + VSL(avg) + WOB(avg) + STR(avg) + LIN(low) + LIN(med) + VAP(med) + VAP(high) + VCL(med) + VCL(high) + WOB(med) + WOB(high) + STR(low) + STR(high))*** | | | | | | | |
| **Coefficient** | ***p*** | **β** |  | **Coefficient** | ***p*** | **β** |  |
| Intercept | **<0.001** | 0.000 |  | *VAP(med)* | **<0.001** | -2.958 |  |
| Sperm conc. | **<0.001** | -0.458 |  | *VAP(high)* | **<0.001** | -13.531 |  |
| VCL(average) | **<0.001** | 7.112 |  | *VCL(med)* | **0.025** | -1.347 |  |
| VSL(average) | **<0.001** | -7.381 |  | *VCL(high)* | 0.156 | 4.624 |  |
| WOB(average) | **0.029** | -1.274 |  | *WOB(med)* | **0.003** | 4.049 |  |
| STR(average) | **<0.001** | 3.449 |  | *WOB(high)* | **<0.001** | 7.344 |  |
| LIN(low) | **0.010** | -1.925 |  | *STR(low)* | **<0.001** | 5.264 |  |
| *LIN(med)* | **0.038** | -1.347 |  | *STR(high)* | **0.028** | -0.513 |  |
| ***Heliocidaris erythrogramma*** | | | | | | | |
| ***Model: lm(Fertilisation ~Sperm concentration + VCL(avg) + LIN(avg) + WOB(avg) + LIN(low) + LIN(high) + VAP(high) + VCL(med) + VCL(high) + VSL(med) + VSL(high) + WOB(low) + WOB(med))*** | | | | | | | |
| **Coefficient** | ***p*** | **β** |  | **Coefficient** | ***p*** | **β** |  |
| *Intercept* | 0.332 | 0.000 |  | *VAP(high)* | **<0.001** | -2.687 |  |
| *Sperm conc.* | **0.010** | -0.320 |  | *VCL(med)* | **<0.001** | 4.081 |  |
| VCL(average) | **<0.001** | -5.127 |  | *VCL(high)* | 0.095 | 2.373 |  |
| *LIN(average)* | **<0.001** | -6.663 |  | *VSL(med)* | **<0.001** | -2.688 |  |
| *WOB(average)* | **0.008** | 1.919 |  | *VSL(high)* | **0.027** | 3.235 |  |
| *LIN(low)* | **<0.001** | -6.790 |  | *WOB(low)* | 0.091 | 1.102 |  |
| *LIN(high)* | **0.003** | 2.129 |  | *WOB(med)* | **0.003** | 0.975 |  |

VCL = curvilinear velocity of sperm path; VSL = straight-line velocity of sperm path; VAP = average sperm path velocity; LIN = sperm path linearity; STR = sperm path straightness; WOB = side-to-side wobble of sperm head.

**Supplementary Table 6: Data presented by ([30]; Table 2) and used to produce Supplementary Figure 2 (below). Fertilisation success in seawater (pH 8.15) and acidified seawater (pH 7.82) for *Crassostrea gigas*. Each trial indicates data from an individual male. Data is for maximum fertilisation success rate under each condition.**

| **Trial** | **# contributing females** | **Fertilisation success (pH 8.15)** | **Fertilisation success (pH 7.82)** | **Difference in fertilisation success** |
| --- | --- | --- | --- | --- |
| A | 3 | 86 % | 84 % | -2 % |
| B | 4 | 82 % | 88 % | 6 % |
| C | 5 | 89 % | 91 % | 2 % |
| D | 3 | 92 % | 88 % | -4 % |
| E | 4 | 71 % | 60 % | -11 % |
| F | 4 | 55 % | 53 % | -2 % |
| G | 3 | 56 % | 59 % | 3 % |
| H | 3 | 37 % | 46 % | 9 % |
| I | 5 | 29 % | 34 % | 5 % |
| J | 3 | 60 % | 59 % | -1 % |
| K | 3 | 58 % | 66 % | 8 % |
| L | 3 | 63 % | 60 % | -3 % |
| M | 3 | 59 % | 60 % | 1 % |
| N | 3 | 65 % | 64 % | -1 % |
| O | 3 | 57 % | 60 % | 3 % |
| P | 3 | 55 % | 54 % | -1 % |

**Supplementary Figure 1: Change in fertilisation performance ranks in current and OA seawater conditions for (a) *Lytechinus pictus* and (b) *Heliocidaris erythrogramma.***

**
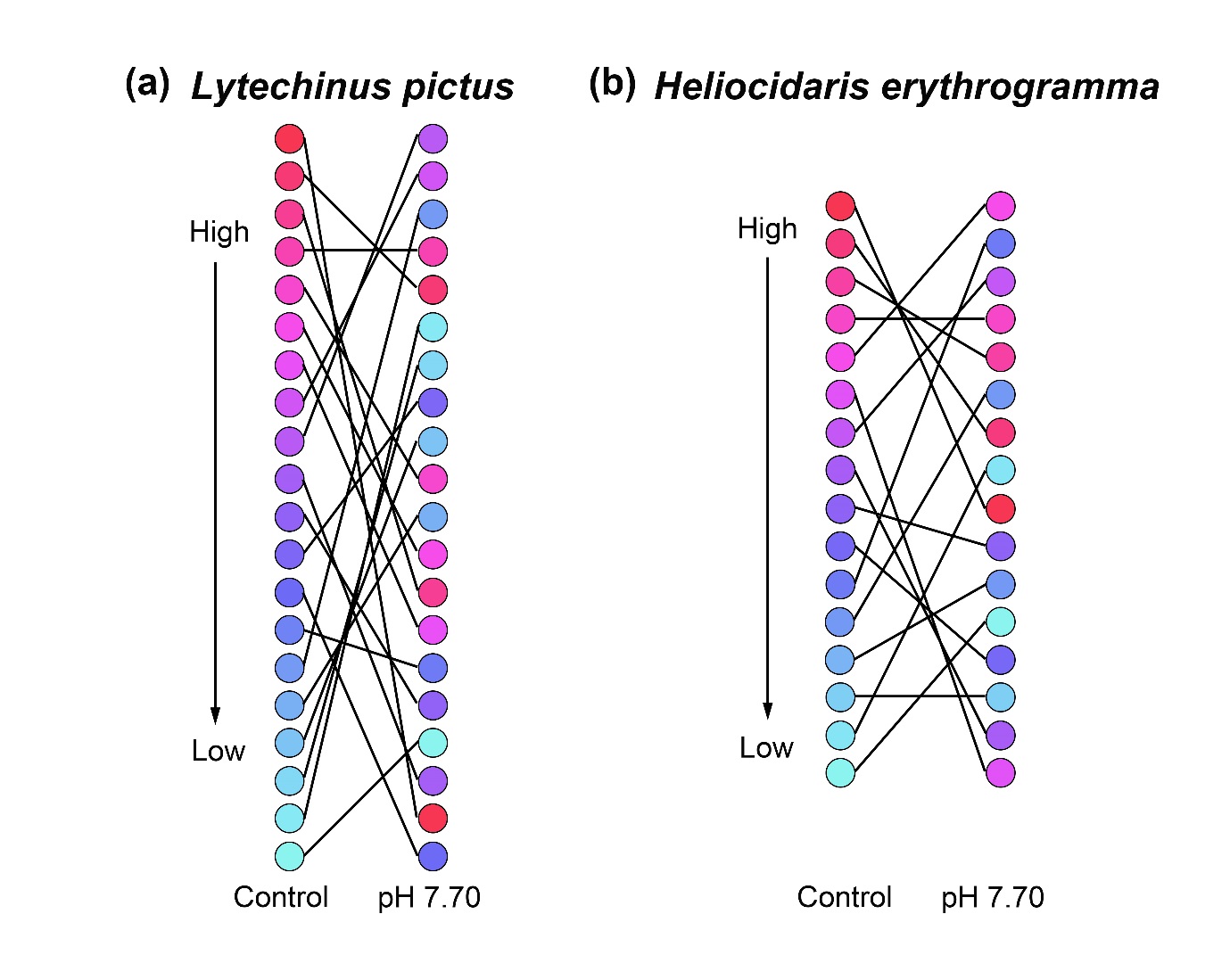
**

**Supplementary Figure 2: Relationship between fertilisation success under control conditions and change in fertilisation success under Ocean Acidification (OA) in *Crassostrea gigas*. Data for fertilisations carried out under control and OA conditions, presented in Table 2 in [30] have been re-analysed (see Supplementary Table 4); control fertilisation rates presented in the table are plotted against difference in fertilisation rate (i.e. the difference between the fertilisation rates for each male under acidified and control conditions). Due to potential problems with gamete compatibility, only trials where multiple females contributed to the eggs used for fertilisation are included. Hence, trials A–P are included but trial Q from the original dataset is excluded. Spearman correlation indicates a significant relationship between fertilisation success under control conditions and change in fertilisation success under acidified conditions (*r_s_* = -0.468, df = 14, *P* = 0.034).**

**
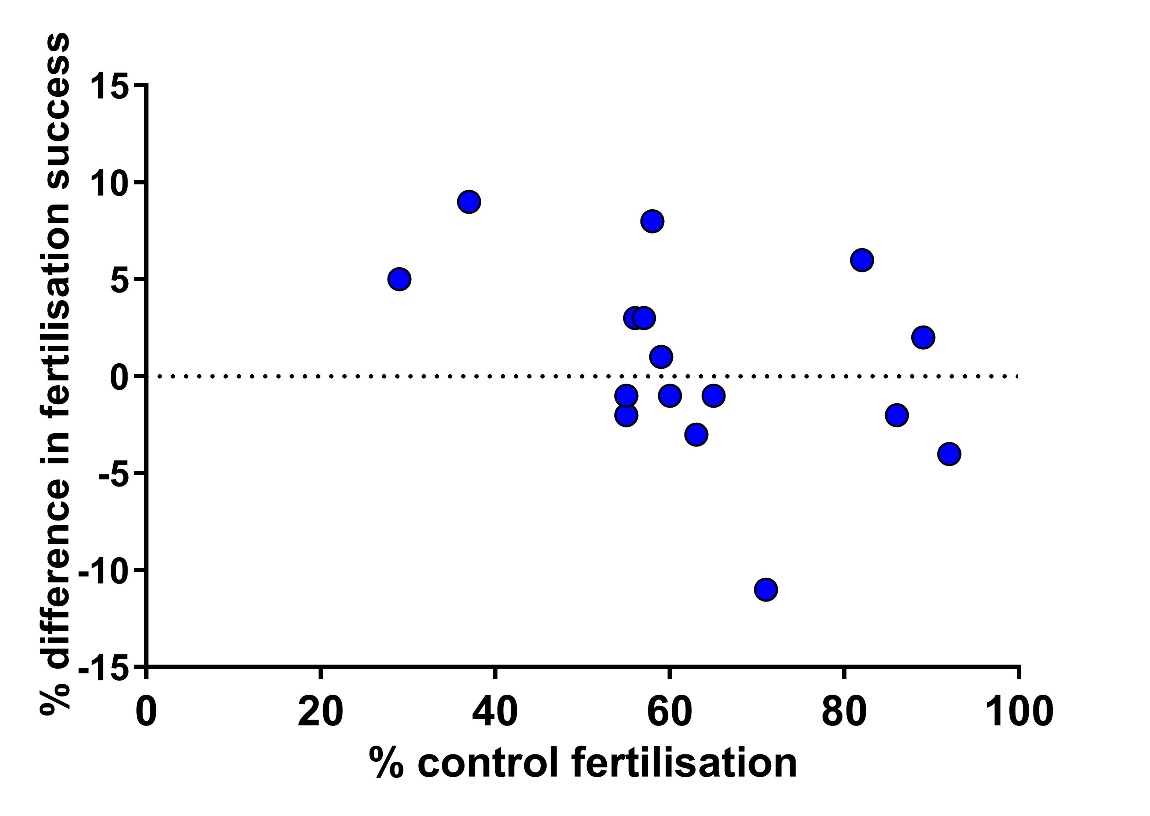
**
