# Supplementary material for: Sea urchin reproductive performance in a changing ocean: poor males improve while good males worsen in response to ocean acidification
Source: Proc Biol Sci. 2019 Jul 24;286(1907):20190785. doi: 10.1098/rspb.2019.0785 (PMC6661356; doi:10.1098/rspb.2019.0785)
Supplement: Reviewer comments [file rspb20190785_review_history.pdf]

THE ROYAL SOCIETY
PUBLISHING

PROCEEDINGS B

Sea urchin reproductive performance in a changing ocean: poor males improve while good males worsen in response to ocean acidification

Kathryn E. Smith, Maria Byrne, Dione Deaker, Cameron M. Hird, Clara Nielson, Alice Wilson-McNeal and Ceri Lewis

Article citation details
Proc. R. Soc. B **286**: 20190785.
http://dx.doi.org/10.1098/rspb.2019.0785

Review timeline

Original submission:	3 April 2019
Revised submission:	15 June 2019
Final acceptance:	28 June 2019

Note: Reports are unedited and appear as submitted by the referee. The review history appears in chronological order.

Review History

RSPB-2019-0785.R0 (Original submission)

Review form: Reviewer 1

Recommendation
Major revision is needed (please make suggestions in comments)

Scientific importance: Is the manuscript an original and important contribution to its field?
Good

General interest: Is the paper of sufficient general interest?
Good

Quality of the paper: Is the overall quality of the paper suitable?
Good

Is the length of the paper justified?
Yes

Reports © 2019 The Reviewers; Decision Letters © 2019 The Reviewers and Editors; Responses © 2019 The Reviewers, Editors and Authors. Published by the Royal Society under the terms of the Creative Commons Attribution License http://creativecommons.org/licenses/by/4.0/, which permits unrestricted use, provided the original author and source are credited

Should the paper be seen by a specialist statistical reviewer?
No

Do you have any concerns about statistical analyses in this paper? If so, please specify them explicitly in your report.
No

It is a condition of publication that authors make their supporting data, code and materials available - either as supplementary material or hosted in an external repository. Please rate, if applicable, the supporting data on the following criteria.

Is it accessible?
Yes

Is it clear?
Yes

Is it adequate?
Yes

Do you have any ethical concerns with this paper?
No

Comments to the Author
In the present work Smith et al. examine the impact of OA on sperm biology and fertilization in 2 ecologically different sea urchin species. Results suggest a significant contribution of inter-individual variability to male fertilization capacity, which is inversely correlated with OA conditions. This manuscript addresses a very important topic (i.e., species reproduction under changing oceans) and the results shed light on the complexity of organismal responses to environmental change and implications for present acclimatization and future adaptation. The manuscript is sound throughout its sections and methods are appropriate as far as the analyses proposed by authors go. That being said, I finished the paper with the feeling that this work left some important aspects behind, specifically when dealing with the mechanisms underlying the responses observed and the complexity of the germ line, not only conveying the genetic instructions to the next generation but also modulating how that information conditions sperm phenotypes and fertilization. Below I highlight some of these aspects which I hope can help authors increase the reach of the work.

P4, L63: when citing sperm phenotypic traits, authors should incorporate references to the nuclear organization of these cells and the impact that sperm nuclear proteins (SNBPs) have on traits relevant for fertilization and sperm competition. A good reference to start is (Ausió, Eirín-López, and Frehlick 2007).

P5, L88: It is not fully clear justified why these two urchin different species have been used as models in the present study. I understand that spawning and other ecological characteristics are clearly different between both of them. However, since this work focuses entirely on sperm phenotypes, I am missing a description of sperm morphology, physiology, nuclear organization, etc in two species.

P6, L125: Experiments where done using male individual sperm and egg pools. However, sperm competition occurs within single individual's ejaculates (across different post-meiotic genetic variants) as well as across sperm from different individuals. I wonder if actual competition (inter-

individual effects) across ejaculates from different individuals would have a significant impact in the motility and fertilization rates of different sperm populations? Is that information lost because of using independent male sperm (not mixed)?

P12, L265: One of the main conclusions of this manuscript is the inverse relationship between fertilization success between current and future OA conditions. Yet, I miss a convincing explanation or hypothesis accounting for this result. I understand that ecological differences between the two urchin species can explain the sperm trait differences observed, and that genetic variation can underlie intra-individual responses seems trivial.

P12, L275: the species name Magallana gigas is NOT correct, please use the name Crassostrea gigas (see more info on this here (Bayne et al. 2019)), this is not negotiable.

P13, L279: Sperm traits can be altered by the genetic outfit of spermatogonia, sperm cells and their development. In addition, these traits can be critically altered by the composition of sperm nuclear basic proteins, affecting hydrodynamic shape and motility, among several other traits (see (Ausió, Eirín-López, and Frehlick 2007)). Importantly, modifications in SNBPs and subsequent impacts on sperm traits constitute potential epigenetic changes that can occur completely (or at least almost completely) independent from genetic alterations, therefore making selective hypotheses more complex. Given the important relevance of these type of modifications for responses to global change (acclimatization, adaptation (Eirin-Lopez and Putnam 2018)), authors should address them throughout the present work.

P14, L314: The work states that inter-individual variability in sperm traits is inherent of male ejaculates. How can that be incorporated into the present discussion of results when sperm fertilization studies were developed individually, without considering potential interactions between sperm cells from different individuals? These inter-individual interactions, as mentioned earlier, are certainly going to modulate sperm traits, including the fertilization ability genetically different sperm populations. I believe this aspect needs to be clearly addressed in order to fully justify the experimental approach followed by this work.

REFERENCES
Ausió, Juan, José Maria Eirín-López, and Lindsay J. Frehlick. 2007. "Evolution of Vertebrate Chromosomal Sperm Proteins: Implications for Fertility and Sperm Competition." Society of Reproduction and Fertility Supplement 65: 63–79.
Bayne, B., M. Anglès d'Auriac, T. Backeljau, P. Beninger, P. Boudry, R. Carnegie, J. Davis, et al. 2019. "A Scientific Name for Pacific Oysters." Aquaculture 499 (January): 373.
Eirin-Lopez, Jose, and Hollie Putnam. 2018. "Marine Environmental Epigenetics." Annual Review of Marine Science in press.

Review form: Reviewer 2

Recommendation
Accept with minor revision (please list in comments)

Scientific importance: Is the manuscript an original and important contribution to its field?
Good

General interest: Is the paper of sufficient general interest?
Good

Quality of the paper: Is the overall quality of the paper suitable?
Excellent

Is the length of the paper justified?
Yes

Should the paper be seen by a specialist statistical reviewer?
No

Do you have any concerns about statistical analyses in this paper? If so, please specify them explicitly in your report.
No

It is a condition of publication that authors make their supporting data, code and materials available - either as supplementary material or hosted in an external repository. Please rate, if applicable, the supporting data on the following criteria.

Is it accessible?
Yes

Is it clear?
Yes

Is it adequate?
Yes

Do you have any ethical concerns with this paper?
No

Comments to the Author
Smith et al. investigated the impacts of ocean acidification on inter-individual variation in fertilization by two species of sea urchin. For both species (Lytechinus pictus and Heliocidaris erythrogramma), near-future conditions altered fertilization success. Individuals with high fertilization success under current conditions exhibited reduced fertilization under near-future conditions (and vice versa). Measuring inter-individual variation and how it may change in the future is more relevant and informative than measuring the "mean response" of the population (as most currently published literature does). The manuscript is well-written, and the experimental design is appropriate and thorough. All my comments are minor, and I have included them below.

Minor comments
Lines 3-4: Is it commonly accepted for ocean acidification to be capitalized, as is done so in the title and running headline?
Line 83: I am assuming the eggs of H. erythrogramma are positively buoyant?
Lines 92-94: Additional supplementary information regarding the in situ ambient pH levels and pH variability these populations are exposed to would be informative. I was unsure whether the values included in Supplementary Table 1 are ambient in situ levels.
Line 119: Were any data collected on sperm longevity? I am curious if there is a trade-off between sperm longevity and other parameters.
Line 199: Did ocean acidification have an effect on the amount of variability found within a population for these sperm functional parameters? Is OA making these parameters more variable or more consistent across the individuals within a population? This information could be pulled

out of this paragraph (and Supplementary Table 2), but it was difficult to interpret as stated.
Line 298: Why do OA conditions select for different sperm swimming parameters? And, what is the mechanism by which OA changes sperm swimming behaviour?
Figure 1G: The percentage of motile sperm observed in L. pictus, even under current conditions (~40%) is lower than I would have expected. Is this data comparable to other studies, or is it indicative that "sub-optimal" sperm (for whatever reason) were used in this experiment? Are the relatively low fertilization rates observed in L. pictus explained by lower sperm concentrations (as indicated in lines 214-217) or actually by less motile sperm?
Discussion: Is there any literature indicating how temperature changes will compound or alleviate inter-individual variation in fertilization success (or sperm swimming parameters)?

Decision letter (RSPB-2019-0785.R0)

17-May-2019

Dear Dr Smith:

Your manuscript has now been peer reviewed and the reviews have been assessed by an Associate Editor. The reviewers' comments (not including confidential comments to the Editor) and the comments from the Associate Editor are included at the end of this email for your reference. As you will see, the reviewers and the Editors have raised some concerns with your manuscript and we would like to invite you to revise your manuscript to address them.

We do not allow multiple rounds of revision so we urge you to make every effort to fully address all of the comments at this stage. If deemed necessary by the Associate Editor, your manuscript will be sent back to one or more of the original reviewers for assessment. If the original reviewers are not available we may invite new reviewers. Please note that we cannot guarantee eventual acceptance of your manuscript at this stage.

To submit your revision please log into http://mc.manuscriptcentral.com/prsb and enter your Author Centre, where you will find your manuscript title listed under "Manuscripts with Decisions." Under "Actions", click on "Create a Revision". Your manuscript number has been appended to denote a revision.

When submitting your revision please upload a file under "Response to Referees" - in the "File Upload" section. This should document, point by point, how you have responded to the reviewers' and Editors' comments, and the adjustments you have made to the manuscript. We require a copy of the manuscript with revisions made since the previous version marked as 'tracked changes' to be included in the 'response to referees' document.

Your main manuscript should be submitted as a text file (doc, txt, rtf or tex), not a PDF. Your figures should be submitted as separate files and not included within the main manuscript file.

When revising your manuscript you should also ensure that it adheres to our editorial policies (https://royalsociety.org/journals/ethics-policies/). You should pay particular attention to the following:

Research ethics:
If your study contains research on humans please ensure that you detail in the methods section whether you obtained ethical approval from your local research ethics committee and gained informed consent to participate from each of the participants.

Use of animals and field studies:
If your study uses animals please include details in the methods section of any approval and licences given to carry out the study and include full details of how animal welfare standards were ensured. Field studies should be conducted in accordance with local legislation; please include details of the appropriate permission and licences that you obtained to carry out the field work.

Data accessibility and data citation:
It is a condition of publication that you make available the data and research materials supporting the results in the article. Datasets should be deposited in an appropriate publicly available repository and details of the associated accession number, link or DOI to the datasets must be included in the Data Accessibility section of the article (https://royalsociety.org/journals/ethics-policies/data-sharing-mining/). Reference(s) to datasets should also be included in the reference list of the article with DOIs (where available).

In order to ensure effective and robust dissemination and appropriate credit to authors the dataset(s) used should also be fully cited and listed in the references.

If you wish to submit your data to Dryad (http://datadryad.org/) and have not already done so you can submit your data via this link http://datadryad.org/submit?journalID=RSPB&manu=(Document not available), which will take you to your unique entry in the Dryad repository.

If you have already submitted your data to dryad you can make any necessary revisions to your dataset by following the above link.

For more information please see our open data policy http://royalsocietypublishing.org/data-sharing.

Electronic supplementary material:
All supplementary materials accompanying an accepted article will be treated as in their final form. They will be published alongside the paper on the journal website and posted on the online figshare repository. Files on figshare will be made available approximately one week before the accompanying article so that the supplementary material can be attributed a unique DOI. Please try to submit all supplementary material as a single file.

Online supplementary material will also carry the title and description provided during submission, so please ensure these are accurate and informative. Note that the Royal Society will not edit or typeset supplementary material and it will be hosted as provided. Please ensure that the supplementary material includes the paper details (authors, title, journal name, article DOI). Your article DOI will be 10.1098/rspb.[paper ID in form xxxx.xxxx e.g. 10.1098/rspb.2016.0049].

Please submit a copy of your revised paper within three weeks. If we do not hear from you within this time your manuscript will be rejected. If you are unable to meet this deadline please let us know as soon as possible, as we may be able to grant a short extension.

Thank you for submitting your manuscript to Proceedings B; we look forward to receiving your revision. If you have any questions at all, please do not hesitate to get in touch.

Best wishes,
Proceedings B
mailto: proceedingsb@royalsociety.org

Associate Editor
Board Member: 1
Comments to Author:

Dear authors,

thank you for your patience with us while we secured peer reviewers for your manuscript. We now have received two reviews that were generally positive about your work. However, the reviewers highlighted several areas that need improvement, mainly regarding the interpretation of the data, before your manuscript can be considered for publication. Please carefully address each of the comments and issues reaised in the manuscript and detail your revisions in a response letter.

Reviewer(s)' Comments to Author:

Referee: 1

Comments to the Author(s)
In the present work Smith et al. examine the impact of OA on sperm biology and fertilization in 2 ecologically different sea urchin species. Results suggest a significant contribution of inter-individual variability to male fertilization capacity, which is inversely correlated with OA conditions. This manuscript addresses a very important topic (i.e., species reproduction under changing oceans) and the results shed light on the complexity of organismal responses to environmental change and implications for present acclimatization and future adaptation. The manuscript is sound throughout its sections and methods are appropriate as far as the analyses proposed by authors go. That being said, I finished the paper with the feeling that this work left some important aspects behind, specifically when dealing with the mechanisms underlying the responses observed and the complexity of the germ line, not only conveying the genetic instructions to the next generation but also modulating how that information conditions sperm phenotypes and fertilization. Below I highlight some of these aspects which I hope can help authors increase the reach of the work.

P4, L63: when citing sperm phenotypic traits, authors should incorporate references to the nuclear organization of these cells and the impact that sperm nuclear proteins (SNBPs) have on traits relevant for fertilization and sperm competition. A good reference to start is (Ausió, Eirín-López, and Frehlick 2007).

P5, L88: It is not fully clear justified why these two urchin different species have been used as models in the present study. I understand that spawning and other ecological characteristics are clearly different between both of them. However, since this work focuses entirely on sperm phenotypes, I am missing a description of sperm morphology, physiology, nuclear organization, etc in two species.

P6, L125: Experiments where done using male individual sperm and egg pools. However, sperm competition occurs within single individual’s ejaculates (across different post-meiotic genetic variants) as well as across sperm from different individuals. I wonder if actual competition (inter-individual effects) across ejaculates from different individuals would have a significant impact in the motility and fertilization rates of different sperm populations? Is that information lost because of using independent male sperm (not mixed)?

P12, L265: One of the main conclusions of this manuscript is the inverse relationship between fertilization success between current and future OA conditions. Yet, I miss a convincing explanation or hypothesis accounting for this result. I understand that ecological differences between the two urchin species can explain the sperm trait differences observed, and that genetic variation can underlie intra-individual responses seems trivial.

P12, L275: the species name Magallana gigas is NOT correct, please use the name Crassostrea gigas (see more info on this here (Bayne et al. 2019)), this is not negotiable.

P13, L279: Sperm traits can be altered by the genetic outfit of spermatogonia, sperm cells and their development. In addition, these traits can be critically altered by the composition of sperm nuclear basic proteins, affecting hydrodynamic shape and motility, among several other traits (see (Ausió, Eirín-López, and Frehlick 2007)). Importantly, modifications in SNBPs and subsequent impacts on sperm traits constitute potential epigenetic changes that can occur completely (or at least almost completely) independent from genetic alterations, therefore making selective hypotheses more complex. Given the important relevance of these type of modifications for responses to global change (acclimatization, adaptation (Eirin-Lopez and Putnam 2018)), authors should address them throughout the present work.

P14, L314: The work states that inter-individual variability in sperm traits is inherent of male ejaculates. How can that be incorporated into the present discussion of results when sperm fertilization studies were developed individually, without considering potential interactions between sperm cells from different individuals? These inter-individual interactions, as mentioned earlier, are certainly going to modulate sperm traits, including the fertilization ability genetically different sperm populations. I believe this aspect needs to be clearly addressed in order to fully justify the experimental approach followed by this work.

REFERENCES
Ausió, Juan, José Maria Eirín-López, and Lindsay J. Frehlick. 2007. "Evolution of Vertebrate Chromosomal Sperm Proteins: Implications for Fertility and Sperm Competition." Society of Reproduction and Fertility Supplement 65: 63–79.
Bayne, B., M. Anglès d'Auriac, T. Backeljau, P. Beninger, P. Boudry, R. Carnegie, J. Davis, et al. 2019. "A Scientific Name for Pacific Oysters." Aquaculture 499 (January): 373.
Eirin-Lopez, Jose, and Hollie Putnam. 2018. "Marine Environmental Epigenetics." Annual Review of Marine Science in press.

Referee: 2

Comments to the Author(s)
Smith et al. investigated the impacts of ocean acidification on inter-individual variation in fertilization by two species of sea urchin. For both species (Lytechinus pictus and Heliocidaris erythrogramma), near-future conditions altered fertilization success. Individuals with high fertilization success under current conditions exhibited reduced fertilization under near-future conditions (and vice versa). Measuring inter-individual variation and how it may change in the future is more relevant and informative than measuring the "mean response" of the population (as most currently published literature does). The manuscript is well-written, and the experimental design is appropriate and thorough. All my comments are minor, and I have included them below.

Minor comments
Lines 3-4: Is it commonly accepted for ocean acidification to be capitalized, as is done so in the title and running headline?

Line 83: I am assuming the eggs of H. erythrogramma are positively buoyant?
Lines 92-94: Additional supplementary information regarding the in situ ambient pH levels and pH variability these populations are exposed to would be informative. I was unsure whether the values included in Supplementary Table 1 are ambient in situ levels.
Line 119: Were any data collected on sperm longevity? I am curious if there is a trade-off between sperm longevity and other parameters.
Line 199: Did ocean acidification have an effect on the amount of variability found within a population for these sperm functional parameters? Is OA making these parameters more variable or more consistent across the individuals within a population? This information could be pulled out of this paragraph (and Supplementary Table 2), but it was difficult to interpret as stated.
Line 298: Why do OA conditions select for different sperm swimming parameters? And, what is the mechanism by which OA changes sperm swimming behaviour?
Figure 1G: The percentage of motile sperm observed in L. pictus, even under current conditions (~40%) is lower than I would have expected. Is this data comparable to other studies, or is it indicative that "sub-optimal" sperm (for whatever reason) were used in this experiment? Are the relatively low fertilization rates observed in L. pictus explained by lower sperm concentrations (as indicated in lines 214-217) or actually by less motile sperm?
Discussion: Is there any literature indicating how temperature changes will compound or alleviate inter-individual variation in fertilization success (or sperm swimming parameters)?

Author's Response to Decision Letter for (RSPB-2019-0785.R0)

See Appendix A.

Decision letter (RSPB-2019-0785.R1)

28-Jun-2019

Dear Dr Smith

I am pleased to inform you that your manuscript entitled "Sea urchin reproductive performance in a changing ocean: poor males improve while good males worsen in response to ocean acidification" has been accepted for publication in Proceedings B.

You can expect to receive a proof of your article from our Production office in due course, please check your spam filter if you do not receive it. PLEASE NOTE: you will be given the exact page length of your paper which may be different from the estimation from Editorial and you may be asked to reduce your paper if it goes over the 10 page limit.

If you are likely to be away from e-mail contact please let us know. Due to rapid publication and an extremely tight schedule, if comments are not received, we may publish the paper as it stands.

If you have any queries regarding the production of your final article or the publication date please contact procb_proofs@royalsociety.org

Open Access
You are invited to opt for Open Access, making your freely available to all as soon as it is ready for publication under a CCBY licence. Our article processing charge for Open Access is £1700. Corresponding authors from member institutions (http://royalsocietypublishing.org/site/librarians/allmembers.xhtml) receive a 25% discount to these charges. For more information please visit http://royalsocietypublishing.org/open-access.

Your article has been estimated as being 10 pages long. Our Production Office will be able to confirm the exact length at proof stage.

Paper charges
An e-mail request for payment of any related charges will be sent out after proof stage (within approximately 2-6 weeks). The preferred payment method is by credit card; however, other payment options are available

Electronic supplementary material:
All supplementary materials accompanying an accepted article will be treated as in their final form. They will be published alongside the paper on the journal website and posted on the online figshare repository. Files on figshare will be made available approximately one week before the accompanying article so that the supplementary material can be attributed a unique DOI.

Thank you for your fine contribution. On behalf of the Editors of the Proceedings B, we look forward to your continued contributions to the Journal.

Sincerely,

Dr Daniel Costa
Editor, Proceedings B
mailto: proceedingsb@royalsociety.org

Associate Editor:
Comments to Author:
Dear authors, thank you for your careful revision of the manuscript. I am delighted to recommend your work for publication in the Proceedings of the Royal Society.

With best regards,

Iliana Baums

Appendix A

BIOSCIENCES

College of Life and Environmental Sciences,
University of Exeter
Geoffrey Pope, Stocker Road,
Exeter. EX4 4QD.

Tel: + 44 (0)1392 263782
E-mail: C.N.Lewis@exeter.ac.uk

15th June 2019

Prof Spencer Barrett
Editor-In-Chief
Proceedings of the Royal Society of London B.

Ref: Sea urchin reproductive performance in a changing ocean: poor males improve while good males worsen in response to ocean acidification

Manuscript ID: RSPB-2019-0785

Authors: Kathryn E. Smith, Maria Byrne, Dione Deaker, Cameron M. Hird, Clara Nielson, Alice Wilson-McNeal, Ceri Lewis

Dear Prof Barrett,

Thank you very much for the helpful and constructive reviewers' comments on the above manuscript.

Both Reviewers agree on the novelty and importance of our work, with Reviewer 1 commenting that it "addresses a very important topic (i.e., species reproduction under changing oceans) and the results shed light on the complexity of organismal responses to environmental change and implications for present acclimatization and future adaptation."

The main comments from Reviewer 1 refer to our design using individual ejaculates and a lack of discussion of the potential mechanisms for the results that we observed. Our aim was to look at how ocean acidification influences individual male reproductive performance by examining how individual sperm phenotypic traits varied with seawater chemistry and how this influenced the fertilisation process under non-competitive scenarios. We have changed the title of our manuscript to make this context clear. We have also revised the manuscript to make the aim clear and to address Reviewer 1's comment about our non-competitive design.

Reviewer 1 also commented about the importance of sperm nuclear proteins as shown largely in the mammalian literature. While our study did not investigate biochemical or genetic mechanisms, we appreciate this insight and have added a paragraph to the discussion to discuss potential molecular mechanisms that might explain our findings, whilst being mindful of the page limit for this publication.

We have fully addressed the comments of both Reviewers. Our full response to each of the points raised and the text changes are detailed below. We feel that we have fully addressed all of the Reviewer's comments. Given that both reviewer's commented on the importance of our research

question, we hope that you will now consider this manuscript for publication. We thank you for your time in managing this manuscript.

Yours sincerely,

Dr Katie Smith

<u>**Reviewer #1**</u>

1. **P4, L63: when citing sperm phenotypic traits, authors should incorporate references to the nuclear organization of these cells and the impact that sperm nuclear proteins (SNBPs) have on traits relevant for fertilization and sperm competition. A good reference to start is (Ausió, Eirín-López, and Frehlick 2007).** We thank the reviewer for highlighting this work and now cite Ausió et al. and comment briefly on the potential role of SNBPs in our observations. However, we note that Ausió et al (2007) is about vertebrate SNBPs with no information provided for invertebrates. This paper focusses on mutations, SNBPs and specific phosphorylation in human SNBPs and how these modifications can alter sperm morphology, motility, fertility and sperm competitiveness. It is interesting to consider how this might be operating in sea urchins, but we are cautious of referring to these mechanisms since there is little to no information on this for invertebrates. We feel it best to avoid speculation but have added some cautious discussion of these points at <u>lines 291-299</u>.
2. **P5, L88: It is not fully clear justified why these two urchin different species have been used as models in the present study. I am missing a description of sperm morphology, physiology, nuclear organization, etc in two species.** Our main rationale for picking these two species was actually their differing egg morphology leading to different fertilisation ecologies, with *Lytechinus* producing the typical small negatively buoyant eggs and *Heliocidaris* producing larger positively buoyant (i.e. floating) eggs. These different egg characteristics would be predicted to place different selection pressures on sperm traits to maximise fertilisation and hence OA might induce differing alterations in sperm function and male fitness accordingly (<u>see lines 81-83</u>). There are also marked differences in the sperm morphology of the two species and we have added new text on this with a relevant citation (<u>lines 84-88)</u>.
3. **P6, L125: Experiments were done using male individual sperm and egg pools. However, sperm competition occurs within single individual's ejaculates (across different post-meiotic genetic variants) as well as across sperm from different individuals. I wonder if actual competition (inter-individual effects) across ejaculates from different individuals would have a significant impact in the motility and fertilization rates of different sperm populations? Is that information lost because of using independent male sperm (not mixed)?** The reviewer's point here is valid and there are numerous experimental designs that could have been used to look at various different aspects of OA effects on fertilisation ecology, all valid according to the specific question being addressed. While this was not the question addressed in our manuscript, we mention this as important to consider <u>(lines 135-136 and 344-346</u>). We designed our experiment to quantify sperm traits based on individual male source as the independent replicate. We have been careful throughout the manuscript to be specific about the question being addressed.
4. **P12, L265: One of the main conclusions of this manuscript is the inverse relationship between fertilization success between current and future OA conditions. Yet, I miss a convincing explanation or hypothesis accounting for this result.** We have added a new paragraph briefly

discussing a number of potential hypothesis for a mechanisms that might explain our findings. We suggest that there may be individual variability in the optimum pH for sperm performance, related to the events leading up to activation (lines 291-299).

5. **P12, L275: the species name Magallana gigas is NOT correct, please use the name Crassostrea gigas (see more info on this here (Bayne et al. 2019)), this is not negotiable.** We have changed this as requested.

6. **P13, L279: Sperm traits can be critically altered by the composition of sperm nuclear basic proteins, affecting hydrodynamic shape and motility, among several other traits (see (Ausió, Eirín-López, and Frehlick 2007)). Importantly, modifications in SNBPs and subsequent impacts on sperm traits constitute potential epigenetic changes that can occur completely (or at least almost completely) independent from genetic alterations, therefore making selective hypotheses more complex. Given the important relevance of these type of modifications for responses to global change authors should address them throughout the present work.** We thank the reviewer for highlighting this work. We have now added references to the Ausió et al. paper (which is vertebrate specific) and mention the importance of the 'testis' environment that sperm develop in which is now emerging as being important for epigenetics in the new paragraph added (see above response). We have struggled to find any literature that provides enough details on how these mechanisms act at the individual level for invertebrates, which is what would be required to be able to link these mechanisms to our observations looking at individual differences in response. We have added a few sentences to the introduction and discussion highlighting that the developmental environment may influence sperm traits (lines 62-63, 291-299) and in turn may have an epigenetic influence, but we have avoided speculation as so little is published for marine invertebrates.

7. **P14, L314: The work states that inter-individual variability in sperm traits is inherent of male ejaculates. How can that be incorporated into the present discussion of results when sperm fertilization studies were developed individually, without considering potential interactions between sperm cells from different individuals? These inter-individual interactions, as mentioned earlier, are certainly going to modulate sperm traits, including the fertilization ability genetically different sperm populations. I believe this aspect needs to be clearly addressed in order to fully justify the experimental approach followed by this work.** The reviewer is correct in saying sperm competition between males will influence fertilisation outcomes but this was not our aim. Our aim in this study was to look at differences in 'effect size' for individual males when examining their non-competitive reproductive performance under two different seawater chemistries. We have been careful to state clearly that we examined individual male responses to pH under non-competitive scenarios (lines 18, 90, 276, 307). To make this context of our manuscript clear, we have changed the title. We have also emphasised throughout the manuscript that our focus is on individual male responses, to make the specific aim of our study much clearer. Finally, we have added a sentence to the discussion indicating that our results could differ under competitive scenarios (lines 344-346).

Reviewer #2

1. **Lines 3-4: Is it commonly accepted for ocean acidification to be capitalized, as is done so in the title and running headline?** We have removed the capitalization as requested.

2. **Line 83: I am assuming the eggs of H. erythrogramma are positively buoyant?** Yes, the eggs are positively buoyant. We have added the word 'positively' to the sentence to clarify this (line 87).

3. **Lines 92-94: Additional supplementary information regarding the in situ ambient pH levels and pH variability these populations are exposed to would be informative. I was unsure whether the values included in Supplementary Table 1 are ambient in situ levels.** For *Heliocidaris*, we have very limited data showing the ambient in situ pH range is approximately 7.95-8.15 (Byrne, unpublished data), although this data is reflective of one week of the year only. There are no data on the pH in the habitat of *Lytechinus*. We have added text to this effect to the legend of Supplementary Table 1.
4. **Line 119: Were any data collected on sperm longevity? I am curious if there is a trade-off between sperm longevity and other parameters.** This is indeed an interesting question and may well occur, but we did not collect data on sperm longevity as part of this study. Importantly, the ten-minute exposure time that we used, is well within the period of an hour or more that sea urchins sperm remain active. Thus, we have no reason to think that sperm longevity influenced our findings. Personal observations confirmed the sperm of both species remained highly active well after the experimental period. We have added details Supplementary Methods, which now reads 'Prior to carrying out fertilisations, sperm activity was tested over an hour to confirm sperm longevity was as is typical of sea urchins[42] and significantly longer than the experimental period we were using sperm over.'
5. **Line 199: Did ocean acidification have an effect on the amount of variability found within a population for these sperm functional parameters? Is OA making these parameters more variable or more consistent across the individuals within a population?** This is indeed an important question. As the reviewer suggests, we have added several sentences to the results detailing how variability across individuals changes within a population under current and OA conditions (lines 191-196).
6. **What is the mechanism by which OA changes sperm swimming behaviour?** As outlined in response to Reviewer 1 (above) we have added text to suggest potential mechanisms explaining why there might be individual variability in the optimum pH for sperm performance (lines 291-299).
7. **Figure 1G: The percentage of motile sperm observed in L. pictus, even under current conditions (~40%) is lower than I would have expected. Is this data comparable to other studies, or is it indicative that "sub-optimal" sperm (for whatever reason) were used in this experiment? Are the relatively low fertilization rates observed in L. pictus explained by lower sperm concentrations (as indicated in lines 214-217) or actually by less motile sperm?** This is a valid comment and something we were initially concerned about. However, to confirm quality control we a) took care to make sure all individuals were very healthy at spawning, and b) spawned individuals on multiple occasions to check % motility was consistent. Upon injection of KCl, all urchins spawned rapidly indicating they were fully gravid.
8. **Is there any literature indicating how temperature changes will compound or alleviate inter-individual variation in fertilization success (or sperm swimming parameters)?** We have looked through the literature examining combined effects of OA and other environmental stressors (temperature, salinity etc), and results indicating what the combined effects are of multiple stressors at an individual level are surprisingly hard to find. The papers we have identified indicate that compounding or alleviating effects vary case-by-case at both the individual-, or population-level. We have added a sentence to the discussion reflecting these findings (lines 356-358).
